# Chronic proliferative rhinitis associated with *Salmonella enterica* subsp. *diarizonae* serovar *61:k:1*,* 5*,* (7)* in a sheep from Romania: First documented case in Southeastern Europe

**DOI:** 10.1007/s11259-026-11430-x

**Published:** 2026-07-25

**Authors:** Adrian-Valentin Potârniche, Emilia Trif, Constantin Cerbu, Emöke Páll, Andrada Negoescu, Cristina Borfălău, Sorin Marian Mârza, Lucia Bel, Florica Bărbuceanu, Marcelo de las Heras, Delia Lacasta, Marian Taulescu

**Affiliations:** 1 Department of Infectious Diseases and Preventive Medicine, Faculty of Veterinary Medicine, University of Agricultural Sciences and Veterinary Medicine of Cluj-Napoca, Mănăstur Str. 3–5, Cluj, 400372 Romania; 2 Department of Anatomic Pathology, Faculty of Veterinary Medicine, University of Agricultural Sciences and Veterinary Medicine of Cluj-Napoca, 3-5 Calea Mănăștur, Cluj- Napoca, 400372 Romania; 3 Department of Clinical Sciences, Faculty of Veterinary Medicine, University of Agricultural Sciences and Veterinary Medicine (UASVM) Cluj-Napoca, 3-5 Mănăştur Street, Cluj-Napoca, 400372 Romania; 4 Department of Surgery Anesthesiology and Intensive Care, Faculty of Veterinary Medicine, University of Agricultural Sciences and Veterinary Medicine, Cluj-Napoca, 400372 Romania; 5 The Institute for Diagnostic and Animal Health (IDSA), Bucharest, Romania; 6 Department of Clinical Sciences II, Faculty of Veterinary Medicine, University of Agronomical Sciences and Veterinary Medicine, Bucharest, 050097 Romania; 7 Department of Animal Pathology, Veterinary Faculty, Instituto Agroalimentario de Aragon- IA2 (Universidad de Zaragoza-CITA), University of Zaragoza, Miguel Servet 177, Zaragoza, 50013 Spain

**Keywords:** Chronic proliferative rhinitis, Romania, *Salmonella enterica* subsp. *diarizonae*, Sheep

## Abstract

A case of chronic proliferative rhinitis (CPR) associated with *Salmonella enterica* subsp. *diarizonae* serovar *61:(k):1*,*5*,*(7)* (SED) is described in a 3-year-old ewe from the university teaching farm in Transylvania, Romania. Clinical examination revealed bilateral seromucous nasal discharge and visible proliferative masses within both nasal cavities. Infrared thermography demonstrated increased heat emission over the nasal region, consistent with active inflammation. Computed tomography imaging showed marked proliferative invasion within the ventral nasal turbinates. Histopathology and immunohistochemistry confirmed chronic proliferative rhinitis characterized by fibrovascular tissue proliferation, epithelial cell hyperplasia, and mixed inflammatory cell infiltrate, predominated by lymphocytes, plasma cells and macrophages. *SED* was isolated as the sole microorganism from nasal swabs and confirmed by serotyping, PCR, and immunohistochemistry. To the authors’ knowledge, this is the first documented case of CPR associated with *SED* in Romania and in Southeastern Europe, highlighting the need to include CPR among the differential diagnoses for adult sheep with chronic upper respiratory disease, and to include SED within its aetiological agents.

**Supplementary Information:**

The online version contains supplementary material available at https://doi.org/10.1007/s11259-026-11430-x.

## Background

Respiratory diseases are widely recognized as significant health issues in major sheep-producing regions (Bell 2008; Scott 2015). These diseases lead to significant economic losses, primarily through increased mortality, decreased productivity, impaired growth, and carcass condemnations at slaughter (Lacasta et al. 2019). Respiratory diseases in small ruminants are multifactorial and encompass a wide range of viral, bacterial, and parasitic agents, many of which contribute to the well-known pulmonary disease complex in sheep and goats (Thompson 2019; Chakraborty et al. 2014; Lacasta et al. 2008). However, beyond these commonly recognized lower respiratory conditions, sheep can also develop a variety of upper respiratory tract diseases, for which the differential diagnosis is broad and complex (Castells et al. 2025; Lacasta and Ruiz 2022).

Chronic proliferative rhinitis (CPR) is an emerging, slow-progressing upper respiratory disease in sheep, characterized by marked mucosal proliferation and chronic inflammation of the nasal turbinates, which may occur unilaterally or bilaterally, and is associated with a poor prognosis in untreated animals (Rubira et al. 2019, 2021). The ventral nasal turbinates consistently represent the primary site of pathology, while involvement of the dorsal turbinate is rarely seen (López-Tamayo et al. 2020). This condition was first described in the United States (Meehan et al. 1992) and has since been reported in other regions of the world, including Spain (López-Tamayo et al. 2020; Figueras et al. 2020; Lacasta et al. 2012) and Switzerland (Stokar-Regenscheit et al. 2017). The disease is typically associated with *Salmonella enterica* subsp. *diarizonae* serovar 61:k:1,5,(7) (SED) (Meehan et al. 1992; Lacasta et al. 2012; Rubira et al. 2019). SED, previously identified as *Salmonella arizonae* (Old 1992), is a motile, Gram-negative, facultatively rod-shaped anaerobic bacterium. It belongs to a subspecies that is frequently isolated from the intestinal microbiota of reptiles (Schröter et al. 2004). SED is, however, considered host-adapted to sheep and was first isolated from sheep in association with abortion cases (Ryff 1952). Besides causing CPR, it has also been associated with stillbirths, diarrhea in lambs, and suppurative epididymo-orchitis in rams (Rubira et al. 2021; Stokar-Regenscheit et al. 2017).

In recent years, this bacterium has gained attention as a pathogen associated with CPR in sheep, evidenced by a rising number of cases described in scientific publications (Figueras et al. 2020; Lacasta et al. 2012; Stokar-Regenscheit et al. 2017).

In all the retrieved documented CPR cases, SED has been isolated as pure growth from the nasal lesions, reinforcing its role as the primary etiological agent of this disease (Rubira et al. 2019). This serovar shows a complex epidemiological behaviour. It has been isolated from tonsils of slaughtered sheep (Bonke et al. 2012) and from nostrils of animals that showed no clinical signs of respiratory disease (Figueras et al. 2020). Moreover, multiple studies have reported rectal spreading of SED in sheep in various countries, without any evidence of associated disease (Methner and Moog 2018; Davies et al. 2001; Alvseike and Skjerve 2002; Sandberg et al. 2002 rén et al. 2015).

These observations suggest that SED is predominantly a commensal in sheep, capable of colonizing the nasal mucosa without inducing disease, but only occasionally inducing to clinically evident CPR (Rubira et al. 2021). Nevertheless, the factors that allow this typically saprophytic bacterium to cross the nasal epithelium and induce CPR remain unknown (Lacasta et al. 2017), although stress is thought to play an important role (Lacasta et al. 2012; Hjartardottir et al. 2002). There are some concerns regarding the zoonotic potential of this bacterium, with immunocompromised individuals and/or those exposed to reptiles considered to be at higher risk (Horvath et al. 2016). To date, there are no documented human cases directly linked to contact with sheep affected by CPR (Rubira et al. 2021).

The objective of this case report is to describe the clinical, microbiological, molecular findings, and the pathology of a CPR case caused by *SED* in a sheep from Romania. To our knowledge, this case represents the first documented occurrence of CPR in the Southestern Europe, expanding the geographic distribution of the disease.

## Case presentation

### Clinical history

A 3-year-old Merino ewe originating from teaching farm of the University of Agricultural Sciences and Veterinary Medicine of Cluj-Napoca (USAMV Cluj-Napoca), located in Cojocna, Transylvania (Romania), was referred to the University Ruminant Clinic of the same institution for clinical evaluation due to respiratory issues. The flock, established from animals purchased from Romanian farms, comprises approximately 300 sheep and 120 cattle raised under extensive production system, with the two species managed separately. Sheep are housed in a concrete building from December to March and are maintained on pasture during the grazing season. The sheep flock is subjected to regular health monitoring, including routine parasitological examinations and systematic vaccination against clostridial diseases and anthrax. According to the farm records and the animal caretakers, the ewe had exhibited gradually worsening respiratory signs over the previous few days, characterized by persistent bilateral seromucous nasal discharge and progressively pronounced stertorous breathing. No other animals in the flock showed comparable respiratory signs at the time of presentation, although occasional coughing had been observed in a few sheep. The ewe had not received any specific treatment for the respiratory condition prior to referral.

### Clinical examination

**Fig. 1:**
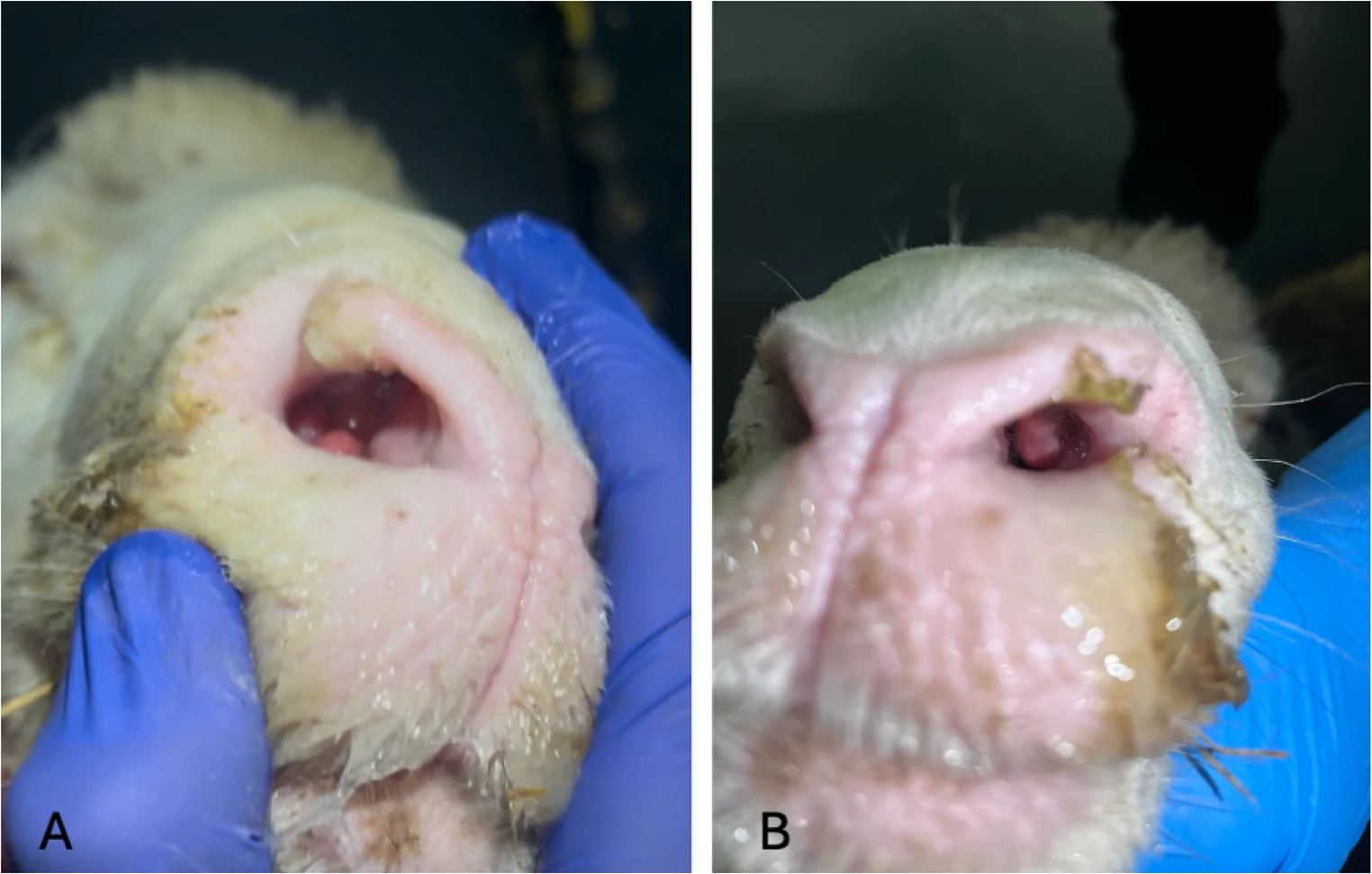
Clinical examination of the nasal cavities in the examined sheep with CPR. Bilateral nasal discharge with intranasal proliferative masses causing obstruction of the right nasal cavity (**A**) and partial obstruction of the left nasal cavity (B)

During the clinical examination, the ewe was alert and in good body condition (body condition score 3/5). Respiratory rate was increased, whereas rectal temperature, heart rate, and other physiological parameters were within normal limits. Respiratory distress was predominantly inspiratory. Intranasal examination revealed proliferative masses within both nasal cavities, resulting in complete obstruction of the right nasal passage (Fig. 1A) and partial obstruction of the left one (Fig. 1B). Thoracic auscultation did not reveal abnormal lung sounds, and no clinical evidence of lower respiratory tract involvement was detected. A subsequent farm visit was conducted to evaluate the overall health status of the flock and to identify additional clinical cases. No other animals presenting comparable upper respiratory abnormalities were observed.

### Diagnostic investigations

Based on the clinical presentation and the intranasal proliferative changes observed during examination, a neoplastic process was initially suspected. Coprological analysis included flotation, sedimentation, and the Baermann technique was performed. No lungworm capable of explaining the respiratory signs were identified. Strongyle-type eggs were detected, with an estimated burden of approximately 200 eggs per gram of feces. Hematological analysis was performed using an automated hematology analyzer (Element HT5+, Antech Diagnostics/Heska, Loveland, CO, USA) and revealed no significant abnormalities.

#### Microbiology and molecular diagnostics

Swabs collected from both nasal cavities were submitted for bacterioscopic and bacteriological examination, including antimicrobial susceptibility testing. Bacterioscopic examination was performed on Gram-stained smears prepared directly from the nasal swabs.

Samples were cultured on selective media, including Xylose Lysine Deoxycholate (XLD) agar and MacConkey agar, and incubated at 37 °C for 48 h. Colonies morphologically consistent with *Salmonella* spp. were subsequently sent to the Institute for Diagnosis and Animal Health (IDSA, Bucharest, Romania) where serotyping was performed by slide agglutination using specific antisera according to the ISO/TR 6579-3:2014 guidelines. In parallel, the isolate was submitted to a reference laboratory in Spain (Exopol, Zaragoza) for polymerase chain reaction (PCR) based molecular confirmation. Both laboratories confirmed the presence of *SED.*

Antimicrobial susceptibility testing was performed using the disk diffusion method (Kirby–Bauer) on Mueller–Hinton agar, following standard laboratory procedures. The isolate was tested against lincomycin, erythromycin, oxytetracycline, sulfamethoxazole–trimethoprim, colistin, tylosin, neomycin, and marbofloxacin.

The highest susceptibility was observed for marbofloxacin, followed by sulfamethoxazole–trimethoprim and tylosin. The isolate demonstrated resistance to the remaining tested antimicrobials.

#### Infrared thermography

**Fig. 2:**
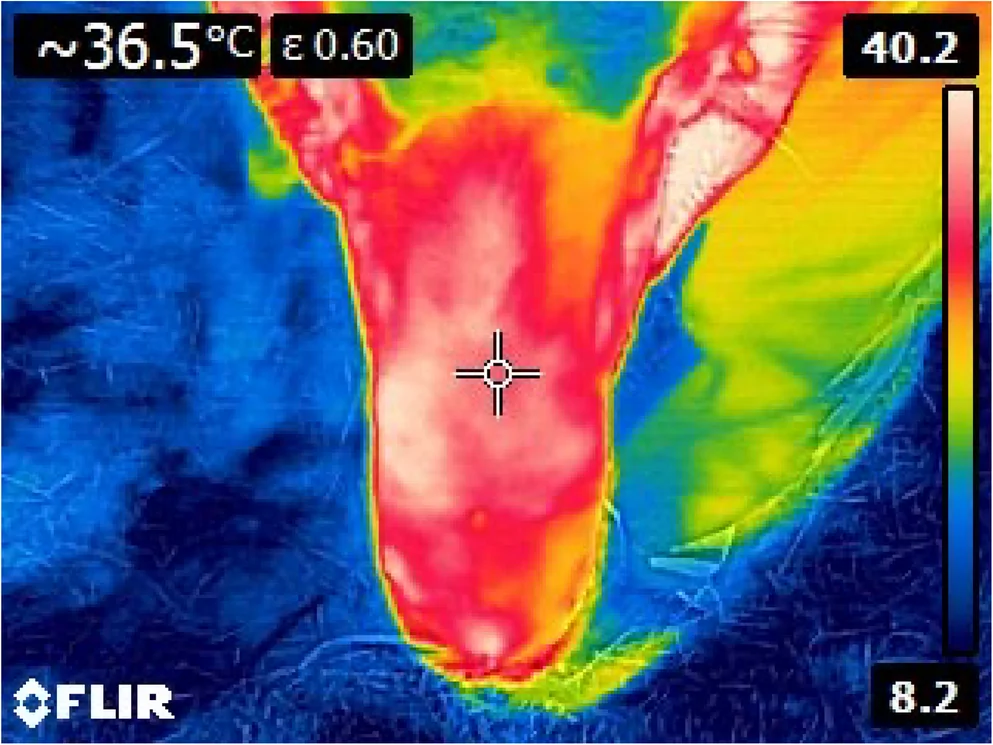
Thermographic findings in the examined sheep with CPR associated with SED. Thermography scan showing bilateral nasal hyperthermia, markedly increased on the right side, consistent with complete airflow obstruction. Red and white colours indicate the highest surface temperatures (hyperthermic areas), whereas green and blue represent progressively lower temperatures

Infrared thermography was used as a complementary diagnostic tool, given its reported utility in the assessment of upper respiratory tract inflammatory processes (Ferrer-López et al. 2020). Thermal imaging was performed using a FLIR E6-XT thermal imaging camera (FLIR Systems Inc., Wilsonville, OR, USA), revealing bilateral hyperthermia of the nasal region, with marked right-sided predominance (Fig. 2), supporting the presence of an active inflammatory process. Based on the thermographic findings in conjunction with the clinical presentation, CPR was included among the differential diagnoses.

#### Computed tomography (CT)

Helical CT of the head was performed using a 16-slice Siemens Somatom Scope scanner. The ewe was positioned in dorsal recumbency under general anesthesia, following premedication with midazolam (0.2 mg/kg IV) and butorphanol (0.1 mg/kg IV), induction with ketamine (2 mg/kg IV) and propofol (2 mg/kg IV to effect). Axial images were acquired using a 512 × 512 matrix, 3 mm slice thickness, and a pitch of 1.5. Images were obtained during the late phase following contrast administration. Multiplanar reconstructions were generated in both soft tissue and bone algorithms at a slice thickness of 1 mm.

**Fig. 3:**
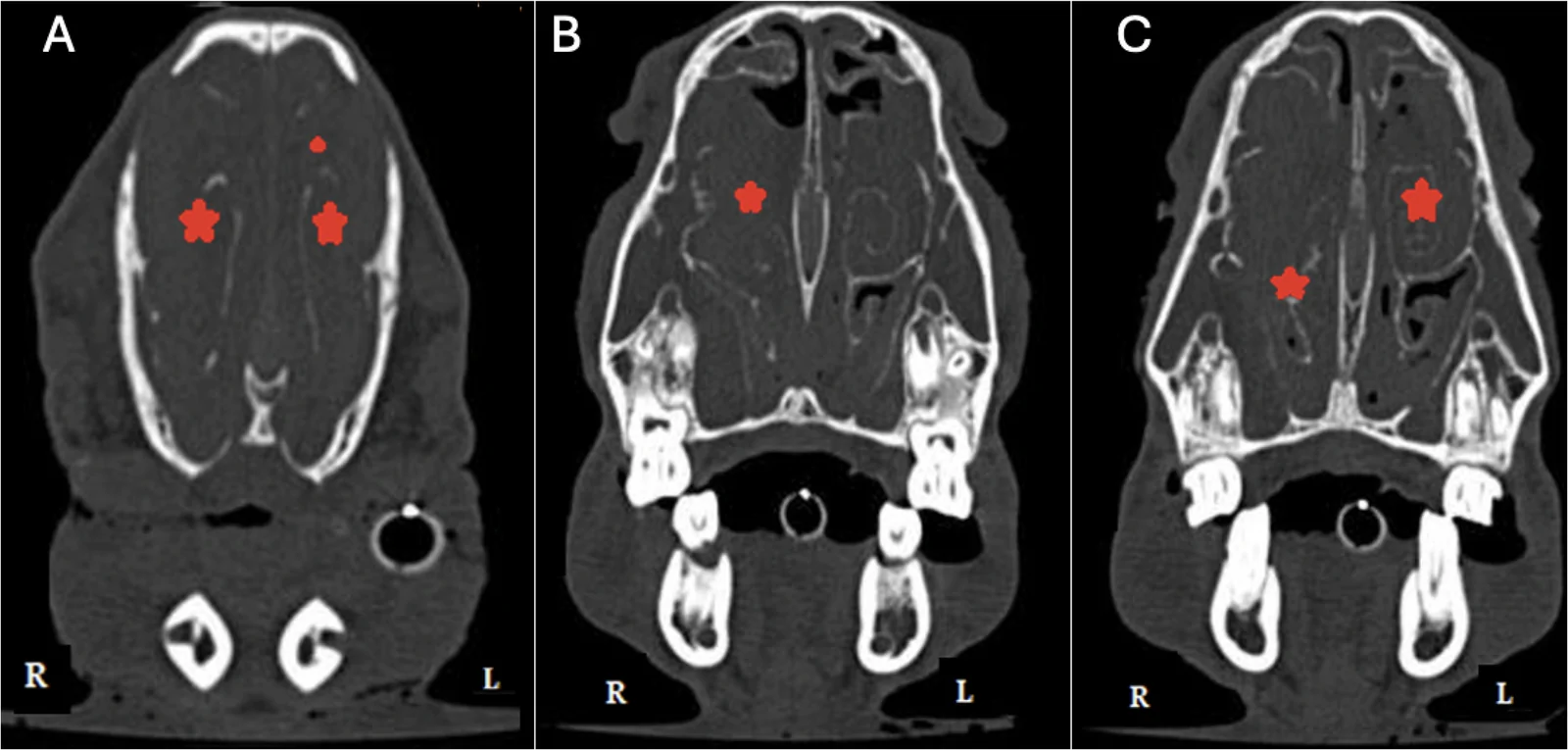
Chronic proliferative rhinitis associated with Salmonella enterica subsp. diarizonae serovar 61:k:1,5,(7) in a sheep. Axial CT images of the nasal cavity. Rostroventral nasal cavity showing bilateral accumulation of fluid-attenuating material within the nostrils (**A**); Mid-nasal section demonstrating partial lysis and remodeling of the right nasal turbinates (**B**); More caudal section showing bilateral fluid accumulation within the nasal passages (**C**). Asteriks indicate the principal CT abnormalities (fluid-attenuating material within the nasal cavity and associated lesions)

**Fig. 4:**
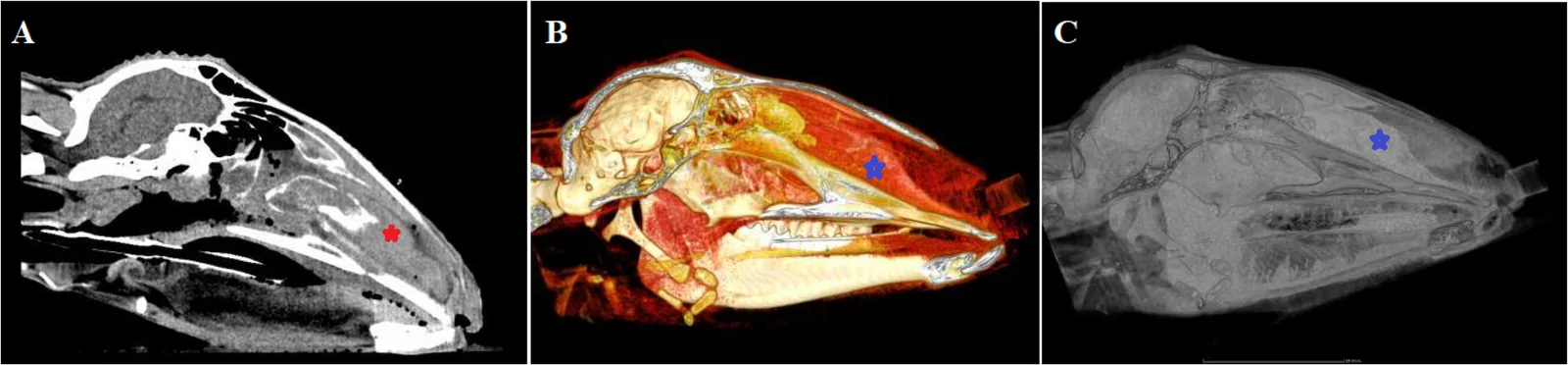
CT findings in a sheep with CPR associated with SED. Sagittal and parasagittal CT images of the head. Rostral nasal cavity showing accumulation of fluid-attenuating material within the nostrils (red asterisk) (**A**); Three-dimensional longitudinal reconstructions demonstrating marked nasal obstruction caused by mucosal thickening and intraluminal fluid (blue asterisk), without evidence of destructive bony changes (**B** and **C**)

CT examination revealed diffuse thickening of the nasal mucosa, with accumulation of fluid-attenuating material within the nasal meatus. The nasal turbinates showed mild atrophy and focal lysis, more pronounced in the middle and ventral areas (Fig. 3). The maxillary sinuses and maxillary recesses contained soft-tissue and fluid-attenuating material, with moderate contrast enhancement of the sinus mucosa. Moderate to severe obstruction of the nasal meatus was observed, particularly in the middle and ventral passages. No significant lysis of the nasal bones was detected (Fig. 4). These findings were consistent with chronic rhinosinusitis characterized by a secretory component and mild turbinate atrophy. The overall imaging pattern was compatible with an inflammatory or infectious process.

#### Rhinoscopy and nasal biopsy

**Fig. 5:**
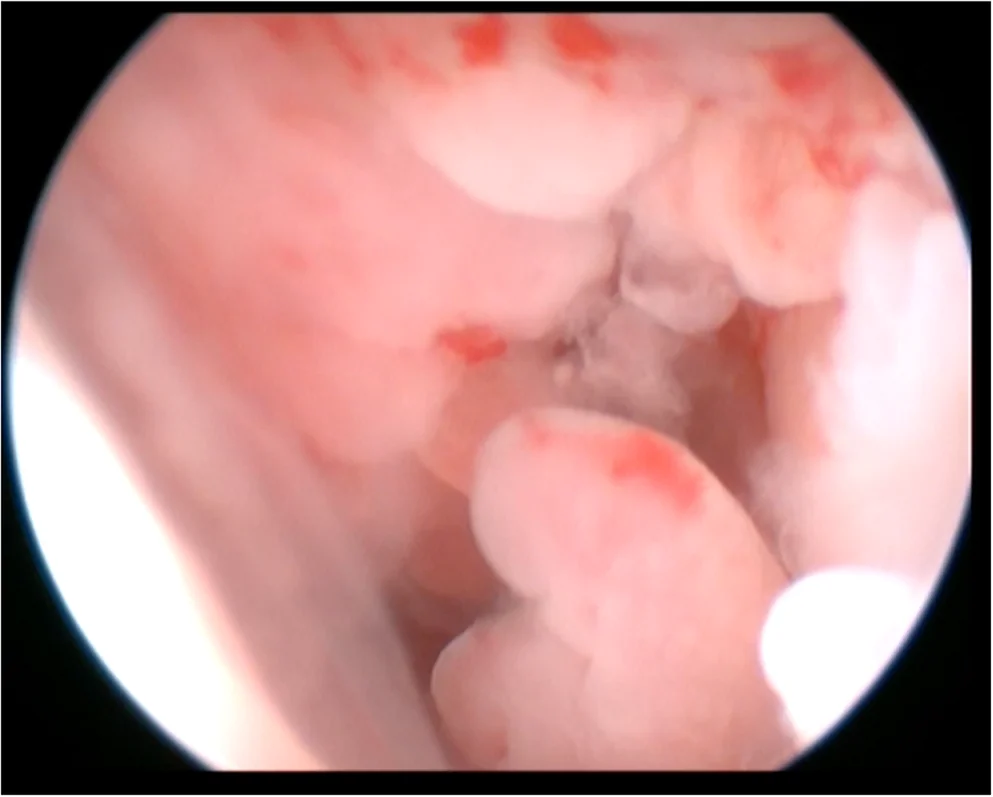
Rhinoscopic findings in a sheep with CPR associated with SED. Endoscopic image of the nasal cavity demonstrating abundant mucous secretion and proliferative mucosal changes with an irregular, polypoid-like appearance consistent with severe narrowing of the nasal lumen

Five days after CT examination and initiation of antimicrobial therapy, under the same general anesthesia protocol described above, rhinoscopy was performed using a 2.7 mm Karl Storz rigid telescope (Karl Storz SE & Co. KG, Tuttlingen, Germany) with a 30^o^ viewing angle and an operative sheath providing a working channel of 3.5 mm outer diameter. Endoscopic examination revealed abundant mucous secretion within both nasal passages, more pronounced on the right side. The mucosa appeared markedly thickened, hyperemic, and irregular with severe luminal obstruction (Fig. 5). During the procedure, tissue specimens were collected from the proliferative lesions within both nasal cavities for pathological investigation. The ewe developed cardiorespiratory arrest after the procedure and died.

#### Postmortem examination

**Fig. 6:**
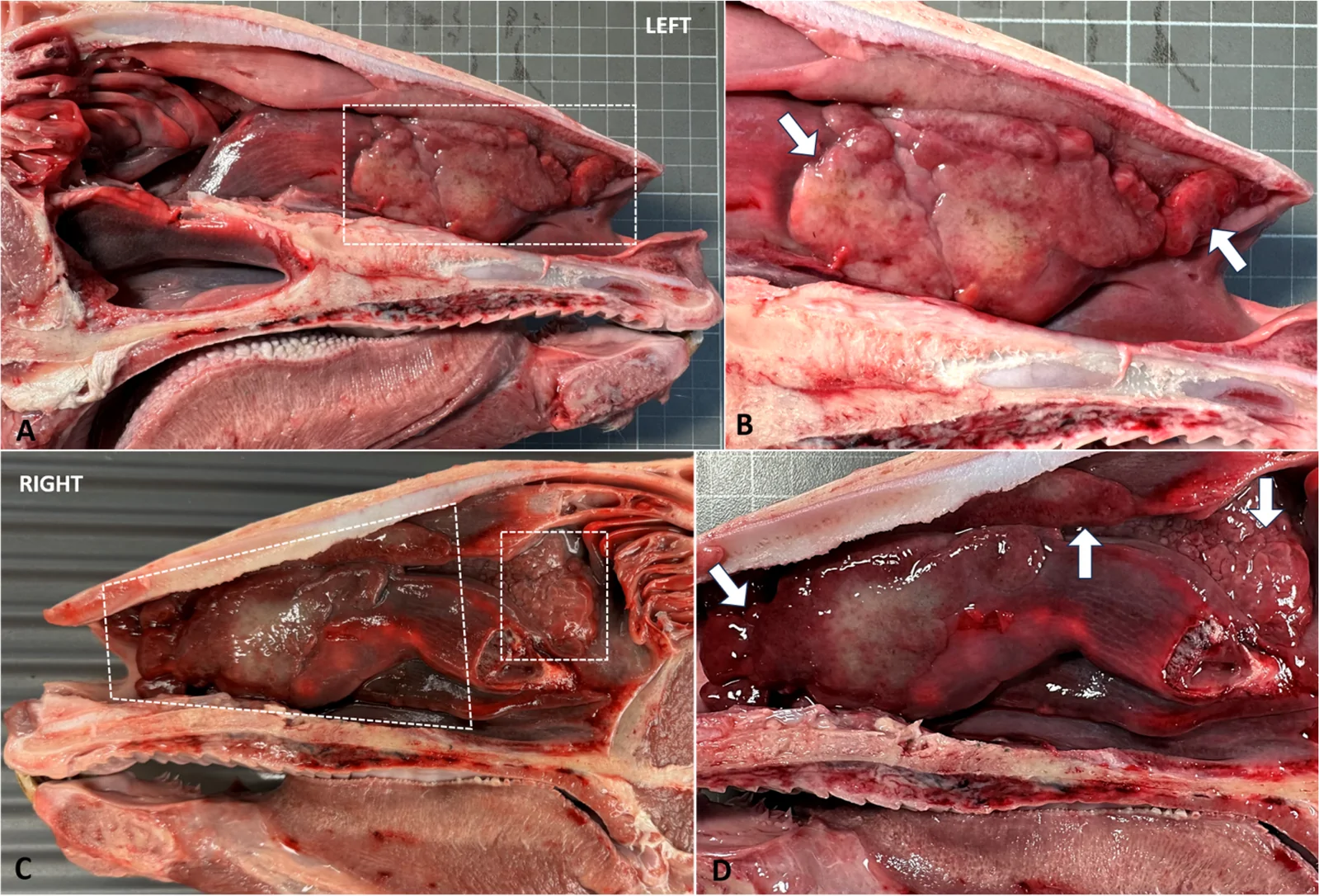
Macroscopical features of chronic proliferative rhinitis in the examined sheep. Parasagittal section of the nasal cavity showing bilateral, proliferative, polypoid-like changes of the nasal mucosa, causing marked narrowing of the nasal passages (left side, delimited area, arrows) (**A** and **B**), more severe on the right side (delimited areas, arrows) (**C** and **D**)

Following skin reflection, the skull was sectioned sagittally along the nasal septum to expose the turbinates, nasal meatuses, and ethmoid region for examination. The nasal mucosa was bilaterally and irregularly thickened, forming multiple firm, gray-pink, coalescing nodular projections that resulted in marked narrowing of the nasal passages. The lesions were more pronounced on the right ventral turbinate (Fig. 6C and D). Small amounts of gray seromucous exudate was present within the nasal meatuses.

#### Histopathology and immunohistochemistry

Tissue samples were fixed in 10% neutral buffered formalin. After paraffin embedding, sections of 2 μm were obtained and stained with hematoxylin and eosin (H&E), Masson Trichrome (MT) and Alcian Blue Periodic Acid-Schiff (ABPAS). Selected paraffin blocks were submitted to the Pathology service of the University of Zaragoza for IHC detection of *Salmonella enterica* subsp. *diarizonae*, while for IHC of cellular markers, the tissue sections were processed within the Department of Anatomic Pathology at USAMV Cluj-Napoca. All reagents and antibodies used for IHC are listed in Table 1.

**Table 1:** Antibodies and reagents used for the IHC procedures

Antibody	Clone	Host species	Supplier	Antigen retrieval and dilution
Vimentin	SRL33	mouse	Leica Biosystems, Newcastle upon Tyne, UK	Ready to use
CK7	RN7			
CD3	LN10			
PAX5	1EW			
Iba1	Polyclonal	rabbit	FUJIFILM Wako Pure Chemical Corporation, Japan	HIER, pH 9, 1:2000
*Salmonella* spp.	MCA-2832	mouse	BioRad	Diluted 1/5000 in TBS

Immunohistochemical staining for vimentin, CD3 and PAX5 was performed using an automated immunostainer (Leica Bond-Max system), with all the reagents being dispensed automatically. For ionized calcium-binding adapter molecule 1 (Iba1), manual processing was conducted with serial dilutions applied after deparaffinization and rehydration. The procedure was performed using a Dako EnVision Flex + kit (mouse, high pH) according to the manufacturer’s instructions, and antigen retrieval was achieved through heat-induced epitope retrieval using Novocastra™ Epitope Retrieval Solution (1:10 dilution) for 30 min. The primary antibody was incubated overnight at 4 °C.

Immunohistochemistry for detection of *Salmonella* spp. was performed using a mouse anti-*Salmonella* antibody (BioRad MCA 2832) and a polymer-based detection system (VECTOR ImmPRESS), following full antigen retrieval and overnight incubation. Following deparaffinization, sections underwent heat-induced antigen retrieval in citrate buffer (pH 6.0), endogenous peroxidase blocking and overnight incubation with the primary antibody at 4 °C. Immunoreactivity was visualized using ImmPACT DAB (Vector Laboratories), and sections were counterstained with hematoxylin. Negative controls were obtained by replacing the primary antibody with normal mouse serum.

The slides were examined with the optical microscope Olympus BX51, and photomicrographs were captured using an Olympus SP350 digital camera and Stream Basic imaging software, version 1.5.1 (Olympus Corporation, Tokyo, Japan).

**Fig. 7:**
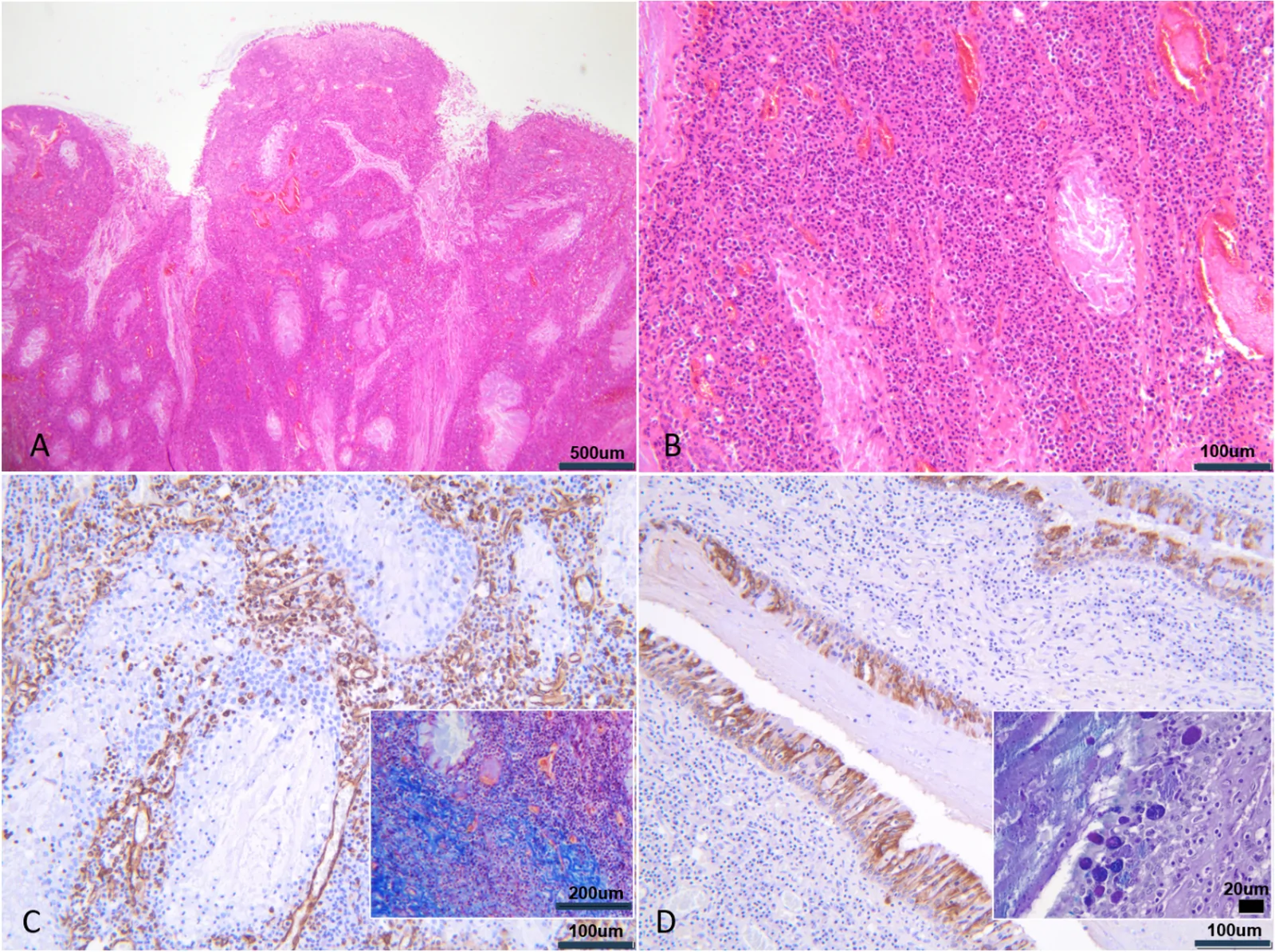
Microscopical findings of CPR associated with SED. The mucosa is irregular and thickened (**A**) due to mononuclear cell infiltrates (**B**), fibrous tissue composed of reactive fibroblasts, blood capillaries and collagen fibers (**C** and inset) and hyperplastic epithelium, containing numerous Goblet cells, and covered by a thick layer of mucus (**D** and inset). **A**, **B** = H&E stain; **C**) IHC for Vimentin (cytoplasmic labeling), the inset - MT stain; **D**) IHC for MCK (cytoplasmic labeling), the inset - ABPAS stain

**Fig. 8:**
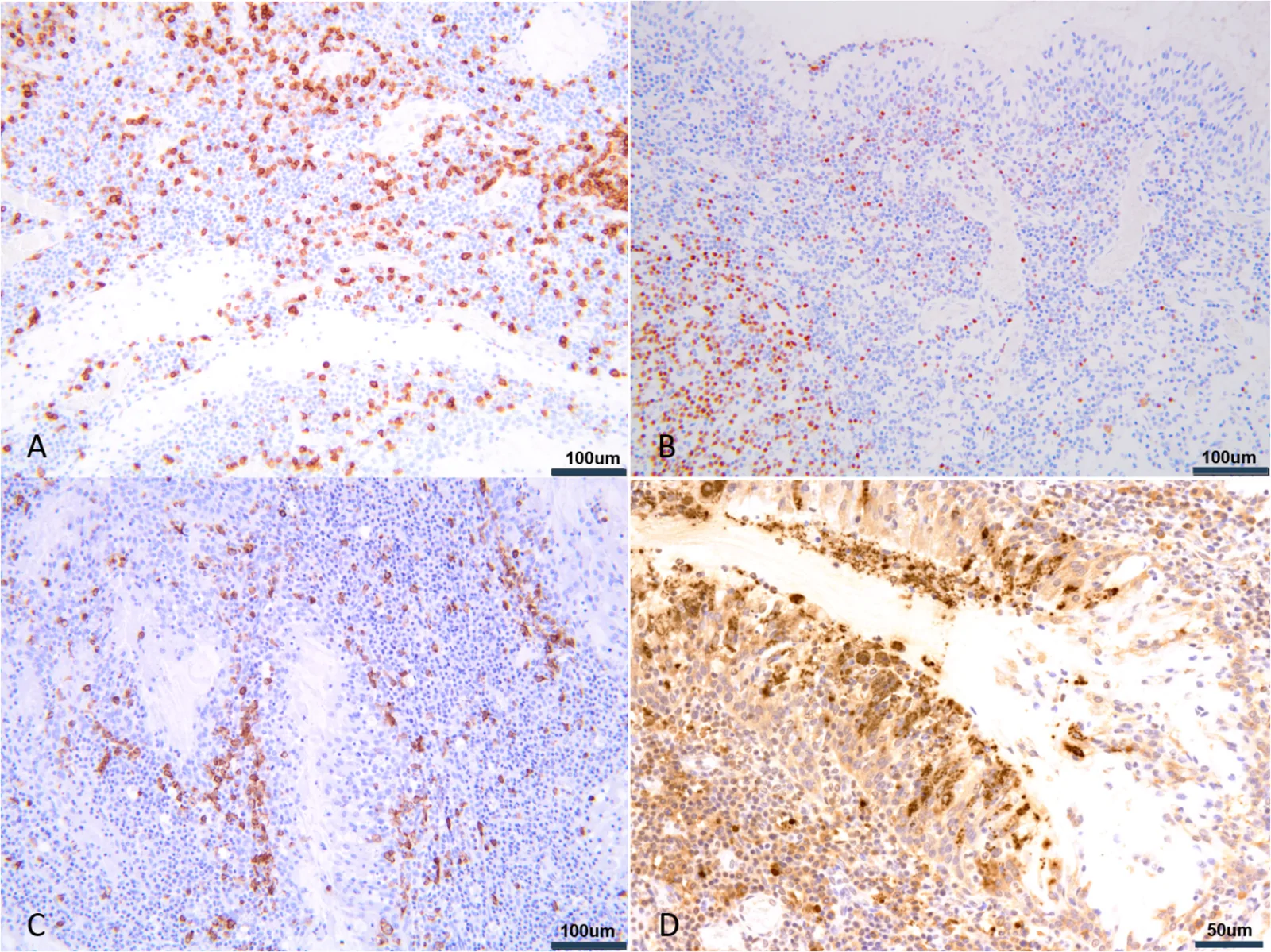
Immunohistochemical characterization of the inflammatory infiltrate and detection of *Salmonella antigen* in the nasal mucosa of a sheep with CPR associated with SED. The inflammatory cells are predominated by CD3+ T (cytoplasmic labeling) (**A**) and PAX5+ B lymphocytes (nuclear labeling) (**B**) and plasma cells, with moderate numbers of IBA1+ macrophages (membranous and cytoplasmic labeling) (**C**). *Salmonella antigen* was detected within hyperplastic epithelial cells and macrophages (**D**)

Microscopical examination showed that the nasal mucosa was diffusely thickened, due to proliferative changes, forming polypoid to finger-like projections (Fig. 7A). The lamina propria was markedly expanded by large numbers of inflammatory cells (Fig. 7B), interspersed with abundant Vimentin positive fibrovascular tissue containing thick and irregular collagen fibers, confirmed by MT stain (Fig. 7C). The overlying epithelium was hyperplastic composed of multiple layers of MCK + epithelial cells, with an increased number of ABPAS+ Goblet cells (Fig. 7D). The nasal glands were also hyperplastic, slightly dilated with mucus and lined by well-differentiated epithelial cells. The inflammatory infiltrates were composed predominantly of CD3 + T and PAX5 + B lymphocytes (Fig. 8A and B), and plasma cells, with moderate numbers of IBA1 + macrophages and rare neutrophils (Fig. 8C). Additionally, immunohistochemistry revealed aggregates of *Salmonella* within hyperplastic epithelial cells, mucoid exudate and macrophages (Fig. 8D).

### Treatment and outcome

Although the proliferative nature of the lesions was not typical for nasal oestrosis, an empirical treatment with ivermectin (Evomec 10 mg/ml, Pasteur) was administered subcutaneously at a dosage of 0.2 mg/kg (1 mL/50 kg). No clinical improvement was observed. Anti-inflammatory treatment with dexamethasone was administered for three consecutive days to reduce mucosal swelling and improve airflow. Based on the antimicrobial susceptibility testing results and in agreement with the available literature for the treatment of CPR (Rubira et al. 2021), antimicrobial therapy with marbofloxacin was initiated at 100 mg/kg once daily for a planned duration of 14 days. On day 5 of treatment, the ewe died developed cardiorespiratory arrest during the rhinoscopy and died.

## Discussion and conclusions

Based on the above findings, a diagnosis of CPR associated with SED was established. This case has important epidemiological implications, as, to the authors’ knowledge, it represents the first documented case of CPR in a sheep from Romania and Southeastern Europe.

*Salmonella* spp. was confirmed through direct bacteriological culture from the inflamed nasal mucosa, while serotyping and PCR specifically identified the isolate as SED, indicating its active presence within the lesions. IHC further demonstrated *Salmonella* antigen within the affected tissue, supporting the direct association between the bacterium and the lesions. Similar findings have been reported previously (Meehan et al. 1992; Lacasta et al. 2012; Stokar-Regenscheit et al. 2017), and the detection of SED both by culture and within the nasal lesions strongly supports its causal involvement in CPR in the present case.

The lesions in this case were in the ventral nasal turbinates, consistent with previous reports (Figueras et al. 2020; Lacasta et al. 2012). So far, only one report has described involvement of the dorsal turbinate and partial ethmoidal extension, which can make clinical diagnosis more challenging (López-Tamayo et al. 2020).

Although SED is considered a commensal of the upper respiratory tract and other tissues, clinical disease is thought to develop only under specific conditions, including stressors such as vaccination, late pregnancy or inappropriate environmental conditions may contribute to disease expression (Lacasta et al. 2012; Brogden et al. 1994; Pritchard 1990). In our case, the ewe was not pregnant, but the flock was kept in overcrowded conditions and had been vaccinated against enterotoxemia a few weeks earlier, factors that may have contributed to the development of clinical disease. The finding indicates that SED is present in Romania and CPR should be considered in the differential diagnosis of upper respiratory disease in adult sheep.

In Europe, the principal differential diagnoses for CPR include oestrosis, enzootic nasal adenocarcinoma (ENA), obstructive rhinopathy associated with pseudopitomycotoxicosis (Lacasta and Ruiz 2022), and, although less commonly reported, inflammatory nasal polyps (Capucchio et al. 2015; Rings and Rojko 1985). In addition, ovine pulmonary adenocarcinoma (OPA) should also be considered, particularly in cases presenting with bilateral nasal discharge (López-Tamayo et al. 2020; Lacasta and Ruiz 2022; Rubira et al. 2019; De Las Heras et al. 2022). Oestrosis typically occurs as a flock-level condition, characterized by inspiratory dyspnea, sneezing, and mucous to purulent or even hemorrhagic nasal discharge resulting from larval migration and mucosal damage (Gracia et al. 2010).

ENA, caused by ENTV-1, is a fatal neoplastic disease that originates from the ethmoidal turbinates and is typically unilateral. Clinical signs include serous unilateral nasal discharge, stertorous breathing, exophthalmos, and cranial deformation as the tumor enlarges (De Las Heras et al. 2003; Walsh et al. 2013). Although ENA was considered due to the inspiratory stertor and nasal obstruction, CT imaging did not reveal a mass arising from the ethmoidal region or evidence of destructive neoplastic invasion.

Obstructive rhinopathy associated with pithomycotoxicosis has been reported in sheep flocks from areas where the fungus Pithomyces chartarum is endemic, particularly in Spain (De Las Heras et al. 2022) and France (Natorp 2005). The condition is associated with facial eczema following the ingestion of sporidesmin-contaminated pastures (Agnetti et al. 2011)and is characterized by partial or complete bilateral nasal obstruction near the nares, with flock-level morbidity that may reach up to 80% (De Las Heras et al. 2022).

Ovine pulmonary adenocarcinoma (OPA) is primarily a lower respiratory tract disease but may occasionally present with nasal discharge, potentially leading to diagnostic confusion (Crilly 2022). In this ewe, thoracic auscultation was unremarkable, and no pulmonary lesions were identified clinically, confirming that the respiratory distress originated from the upper airways.

Other granulomatous conditions of the nasal cavity, including conidiobolomycosis and similar fungal diseases described mainly in South America and Australia, may also produce obstructive proliferative lesions (Carrigan et al. 1992; Portela et al. 2010). However, these conditions are extremely rare in Europe and typically associated with marked granulomatous inflammation and tissue destruction (Rubira et al. 2019).

On the post-mortem examination, the differentiation between these diseases is straightforward. ENA is a unilateral tumor of the ethmoidal region, oestrosis is confirmed by the presence of larvae in the nasal cavity, OPA presents as a distinct lower respiratory tract neoplasm with characteristic pulmonary lesions, and obstructive rhinopathy associated with pithomycotoxicosis is characterized by bilateral narrowing of the nasal lumen near the vestibulum due to whitish obstructive proliferative inflammatory tissue that partially or completely occludes the nasal meatus. CPR, in contrast, is an inflammatory and proliferative process predominantly affecting the ventral turbinates.

Several of the differential diagnoses considered, oestrosis (Mot 2008), ENA (unpublished data) and OPA (Hodor et al. 2024; Toma et al. 2020) have all been previously reported in Romania. It is very likely that CPR has been underdiagnosed until now, either because affected animals were not submitted for advanced diagnostic investigation or because lesions were mistakenly attributed to more familiar conditions such as parasitic or neoplastic rhinitis. It must be considered that this serovar is considered potentially zoonotic, even though no transmission from sheep to humans has been reported to date (Rubira et al. 2021).

Increasing awareness among veterinary practitioners and pathologists is essential to improve recognition of CPR. Prevalence studies and epidemiological surveillance in the area are suggested to determine how widely SED is carried in sheep populations.

## Supplementary Information

Below is the link to the electronic supplementary material.

Supplementary Material 1

Supplementary Material 2

## Data Availability

No datasets were generated or analysed during the current study.
