# Supplementary material for: Chronic proliferative rhinitis associated with Salmonella enterica subsp. diarizonae serovar 61:k:1, 5, (7) in a sheep from Romania: First documented case in Southeastern Europe
Source: Vet Res Commun. 2026 Jul 25;50(5):483. doi: 10.1007/s11259-026-11430-x (PMC13401581; doi:10.1007/s11259-026-11430-x)
Supplement: Supplementary file 2 — Supplementary Material 2 [file 11259_2026_11430_MOESM2_ESM.docx]

**Supplementary Video S1.** Clinical examination of the nasal cavities in a sheep with chronic proliferative rhinitis (CPR). The video demonstrates bilateral nasal discharge and intranasal proliferative masses causing obstruction of the right nasal cavity and partial obstruction of the left nasal cavity.
